# *Shh*edding New Light on the Role of Hedgehog Signaling in Corneal Wound Healing

**DOI:** 10.3390/ijms23073630

**Published:** 2022-03-26

**Authors:** Xin Zhang, Stéphane Mélik-Parsadaniantz, Christophe Baudouin, Annabelle Réaux-Le Goazigo, Nathan Moreau

**Affiliations:** 1 CNRS, INSERM, Institut de la Vision, Sorbonne Université, 17 Rue Moreau, F-75012 Paris, France; xin.zhang@inserm.fr (X.Z.); stephane.melik-parsadaniantz@inserm.fr (S.M.-P.); cbaudouin@15-20.fr (C.B.); annabelle.reaux@inserm.fr (A.R.-L.G.); 2 CHNO des Quinze-Vingts, INSERM-DGOS CIC 1423, 28 Rue de Charenton, F-75012 Paris, France; 3 Department of Ophtalmology, Ambroise Paré Hospital, AP-HP, 9 Avenue Charles de Gaulle, F-92100 Boulogne-Billancourt, France; 4 Laboratory of Orofacial Neurobiology, EA 7543, Université de Paris, 5 Rue Garancière, F-75006 Paris, France

**Keywords:** corneal wound healing, corneal homeostasis, corneal neovascularization, sonic hedgehog, nerve injury, oxidative stress

## Abstract

The cornea, an anterior ocular tissue that notably serves to protect the eye from external insults and refract light, requires constant epithelium renewal and efficient healing following injury to maintain ocular homeostasis. Although several key cell populations and molecular pathways implicated in corneal wound healing have already been thoroughly investigated, insufficient/impaired or excessive corneal wound healing remains a major clinical issue in ophthalmology, and new avenues of research are still needed to further improve corneal wound healing. Because of its implication in numerous cellular/tissular homeostatic processes and oxidative stress, there is growing evidence of the role of Hedgehog signaling pathway in physiological and pathological corneal wound healing. Reviewing current scientific evidence, Hedgehog signaling and its effectors participate in corneal wound healing mainly at the level of the corneal and limbal epithelium, where Sonic Hedgehog-mediated signaling promotes limbal stem cell proliferation and corneal epithelial cell proliferation and migration following corneal injury. Hedgehog signaling could also participate in corneal epithelial barrier homeostasis and in pathological corneal healing such as corneal injury-related neovascularization. By gaining a better understanding of the role of this double-edged sword in physiological and pathological corneal wound healing, fascinating new research avenues and therapeutic strategies will undoubtedly emerge.

## 1. Introduction

**Figure 1:**
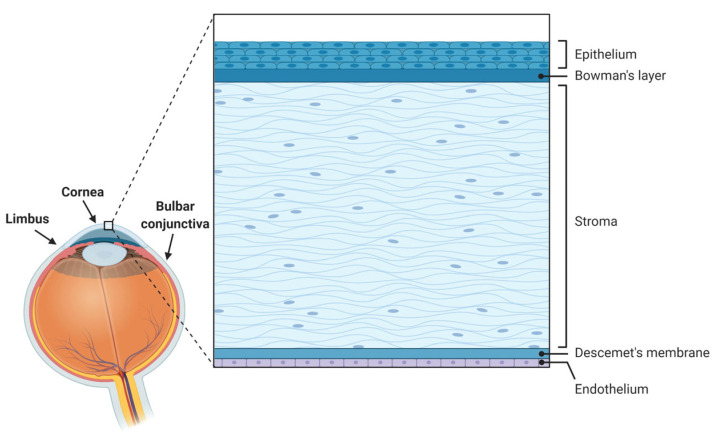
Anatomy of the ocular surface and histology of the human cornea and its various layers.

The cornea is a specialized avascular transparent lens-shaped structure of the anterior segment of the eye that serves to protect the ocular surface from external insults via its epithelium and the tear film and to refract light (providing about two-thirds of the eye’s refractive power) [1]. Comprising five layers in humans [1,2,3] from front to back, namely epithelium, Bowman’s layer, stroma, Descemet’s membrane, and endothelium (Figure 1), the cornea is under constant renewal, especially at the epithelial level. Such corneal epithelial renewal provided by stem cells originating in the limbus [4] allows the maintenance of its functional and structural integrity despite numerous and frequent mechanical, thermal, chemical, or environmental insults [1,5].

Corneal healing, similar to other types of wound healing, is a dynamic and complex process divided into three main phases, namely inflammation (wound detersion), cell proliferation and migration (tissular regeneration), and remodeling (tissular homeostasis restoration) [6]. As the cornea is an avascular tissue, no hemostasis occurs at the early stages of healing (as is the case for other vascularized tissues), but instead, fibrinolysis allows the dissolution of fibrin released following stromal injury and regulation of extracellular matrix turnover during healing [7].

This healing process involves multiple cellular and molecular mechanisms, including cell migration and proliferation under the paracrine control of cytokines and growth factors in the different layers of the cornea [8]. Understanding such mechanisms and underlying molecular pathways is of paramount clinical importance to develop new therapeutic strategies aimed at fostering better, faster corneal healing and restoring ocular surface homeostasis.

Although several key cell populations (limbal stem cells, corneal epithelial cells, corneal fibroblasts, corneal nerves, immunocytes, etc.) and molecular pathways (extracellular matrix components, growth factors, cytokines, etc.) have already been thoroughly investigated, insufficient, impaired, or excessive corneal wound healing remains a major clinical issue in ophthalmology, and new avenues of research are still needed to further improve corneal wound healing [5,6,9].

Considering its major and quasi-ubiquitous role in embryological development, tissue homeostasis, and tissue repair [10], as well as in oxidative stress [11] and cell cycle regulation [12], several studies have started investigating the putative role of the Hedgehog morphogenetic signaling pathway in physiological and pathological corneal wound healing.

This review aims to summarize the current data regarding the implication of the Hedgehog pathway as an effector of corneal wound healing and discuss its relevance as a new avenue for corneal wound healing research.

## 2. A Primer on Corneal Anatomy and Corneal Wound Healing

### 2.1. Corneal Anatomy and Physiology

The corneal epithelium, the outermost layer of the cornea, is a self-renewing non-keratinized stratified squamous epithelium four to six layers thick that undergoes complete turnover in approximately one week [2].

The stroma forms about 80–85% of the cornea’s thickness [2] and consists of keratocytes embedded in a matrix of highly organized collagen fibrils and proteoglycans. Such collagen fibrils are arranged in parallel layers called lamellae, responsible for both the shape of the cornea and some of the transparency of the stroma, critical for vision. Stromal transparency is determined by the near absence of light scattering by keratocytes, the absence of blood vessels, low water content, the absence of pigments, and the size and assembly of collagen fibrils [13]. In that respect, the cornea is a site of immune and angiogenic privilege, which both serve to maintain corneal transparency by preventing light-scattering immunocyte infiltration and blood-vessel formation [6].

**Figure 2:**
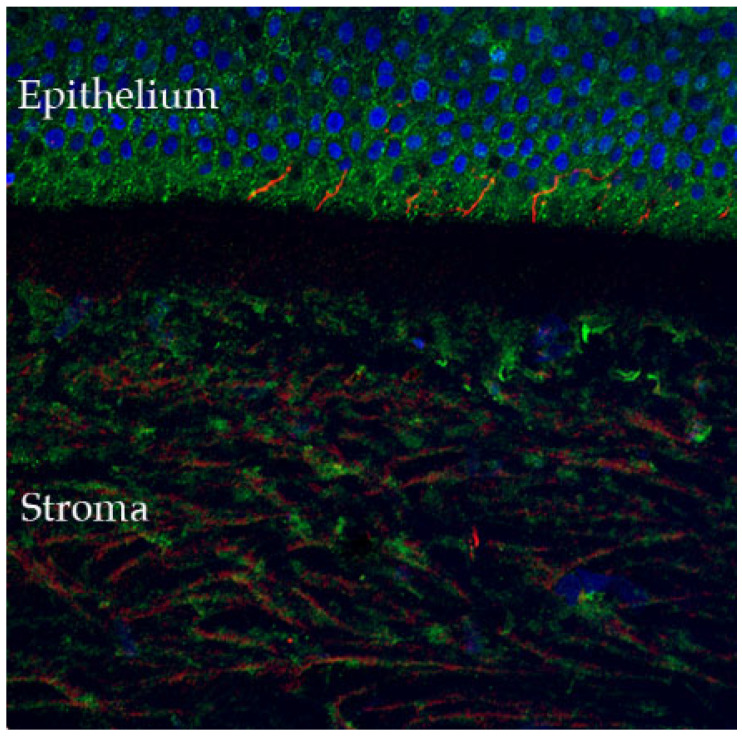
Confocal microscopy-evidenced immunostaining of corneal nerves (in red) in a healthy human cornea sample (×40 magnification). Of note, the corneal fibers that penetrate in the corneal epithelium, where they participate in stromal-epithelial interactions (detailed in Section 2.4) (DAPI (blue) was used to stain the epithelial cell nuclei).

The cornea is the most densely innervated tissue in the human body (estimated 300–600 times more than skin and 20–40 times more than dental pulp [14,15]), receiving sensory innervation from the ophthalmic branch of the trigeminal nerve, with sensory neuron cell bodies located in the dorsomedial portion of the ophthalmic region of the trigeminal ganglion [15,16]. The nerve bundles enter the cornea in the periphery at the level of the corneo-scleral limbal region, forming the limbal plexus, from which stromal nerve trunks emanate and penetrate the cornea in a radial fashion. From there, nerve fibers progress anteriorly where they divide into several small branches, reaching the epithelium from the periphery to the center [15], where they penetrate the first epithelial layers and participate in stromal-epithelial interactions (Figure 2).

Indeed, close and coordinated interactions between epithelium and stroma mediated by growth factors and cytokines are necessary for physiological corneal wound healing. This complex process involves the corneal epithelium, stromal keratocytes, vascular endothelium at the limbus, and inflammatory/lacrimal gland cells, under the neurotrophic influence of corneal nerves [5].

Although the corneal endothelium can be specifically injured and participates in corneal wound healing, most of the studies pertain only to epithelial and stromal mechanisms of wound healing [4] and will thus be the focus of the following sections.

Overall, the major aims of corneal healing are to restore both corneal barrier integrity and stromal determinants of corneal transparency [1].

### 2.2. Epithelial Injury

Injuries limited to the epithelium, with no damage to the basement membrane, will seldom have major repercussions at the stromal level such as stromal haze, also known as corneal fibrosis, scarring, or opacity [17], but can lead to stromal keratocyte apoptosis via epithelial-stromal interactions [1]. Following epithelial injury, a highly regulated inflammatory reaction will occur, leading to limbal stem cell proliferation and migration [4,6] to cover the wound, thus restoring the corneal barrier, at approximately 100 µm per hour (as evidenced in a rabbit cornea model) [18]. Epithelial healing will conclude with the formation of adhesion structures anchoring the regenerated epithelium to the underlying stroma [1].

### 2.3. Stromal Injury

With more severe corneal injury, both the corneal epithelium and stroma may be directly injured. Following stromal injury, keratocytes beneath the injured epithelium undergo immediate apoptosis, whereas some keratocytes also undergo necrosis [19]. Adjacent keratocytes proliferate, migrate, and activate, thus acquiring a fibroblastic phenotype, and finally, transform into myofibroblasts [20]. Both fibroblasts and myofibroblasts secrete a transient matrix scaffold [21], whereas both epithelial and stromal cells trigger a chemokine-mediated inflammatory cell influx allowing the clearance of apoptotic and necrotic debris. Repair and remodeling then occur secondary to the secretion of various metalloproteinases (such as matrix metalloproteinase-2 (MMP-2) or membrane-type 1-MMP (MT1-MMP)) by epithelial cells, fibroblasts, myofibroblasts, and inflammatory cells at the various levels of the cornea [22]. Finally, normal form and function of the stroma are slowly restored following the resorption of the abnormal extracellular matrix and apoptosis of myofibroblasts or reversal of the myofibroblastic phenotype [23].

### 2.4. Stromal-Epithelial Interactions in Corneal Wound Healing

Bidirectional interactions between corneal epithelium and stroma occur during corneal wound healing, implicating numerous signaling pathways [1], several of which are detailed hereafter to illustrate the aforementioned interactions.

The Epidermal Growth Factor (EGF) pathway is responsible for initiating epithelial cell migration and proliferation, in association with Insulin-like Growth Factor (IGF), insulin, Transforming Growth Factor β (TGF-β), and Platelet-Derived Growth Factor (PDGF) [5]. Proinflammatory cytokines/chemokines such as Interleukin 1 (IL-1), C-C Motif Chemokine Ligand 2 (CCL-2), and Tumor Necrosis Factor α (TNF-α) released by epithelial cells lead to apoptosis of underlying keratocytes. Corneal epithelial PDGF leads to the migration, proliferation, and differentiation of keratocytes [1].

Conversely, Hepatocyte Growth Factor (HGF) and Keratinocyte Growth Factor (KGF) secreted by stromal keratocytes modulate the motility, proliferation, and differentiation of corneal epithelial cells [1].

TGF-β, normally restricted to the epithelium in the healthy uninjured cornea, can also be secreted by infiltrating macrophages in the stroma following injury [1], where it plays a role in the differentiation of myofibroblasts from keratocytes. Both activated keratocytes and myofibroblasts then participate in the secretion of the transient matrix consisting of fibronectin, proteoglycans, and hyaluronan [21] as part of the corneal regeneration process [1].

Finally, specific mention should be made of EMMPRIN (Extracellular Matrix MetalloPRoteinase Inducer, CD147) as a quintessential example of a key player in epithelial-stromal interactions during corneal wound healing (reviewed in [24]). EMMPRIN is a 58kD transmembrane glycoprotein expressed at the surface of corneal epithelial cells that promotes MMP expression (notably MMP-1, MMP-2, and MMP-3) in the stroma following a corneal injury via a direct cell–cell interaction with stromal fibroblasts [25]. Furthermore, EMMPRIN is upregulated by TGF-β, where it is responsible for the aforementioned differentiation of stromal keratocytes into myofibroblasts via the induction of α-smooth muscle actin expression and collagen gel contraction [26]. Moreover, by regulating MMP-mediated epithelial tight-junction protein cleavage, EMMPRIN has been shown to modulate the corneal epithelial barrier function in physiological and pathological corneal wound healing such as in dry eye disease [27].

### 2.5. The Two-Faced Role of Corneal Innervation in Corneal Wound Healing

From a pathophysiological standpoint, corneal nerves present a two-faced role in corneal homeostasis. On the one hand, they contribute to the maintenance of corneal integrity by secreting trophic neuropeptides (such as substance P (SP), calcitonin-gene related peptide (CGRP), pituitary adenylate cyclase-activation polypeptide (PACAP), and vasoactive intestinal peptide (VIP) [28]), promoting proper tear secretion and blink reflex [29]. On the other hand, acute and/or chronic dysfunction of corneal nerves can initiate neurogenic inflammation via CGRP/SP-mediated signaling and may eventually lead to corneal opacification and vision impairment. Furthermore, exuberant inflammation in the cornea can intensify corneal nerve dysfunction (neuroinflammatory-induced corneal nerve damage) and can lead to a vicious circle where inflammation-mediated leukocyte infiltration and neovascularization can lead to permanent corneal damage and chronic pain [29,30].

## 3. Hedgehog Signaling: A Forgotten Player in Corneal Wound Healing?

**Figure 3:**
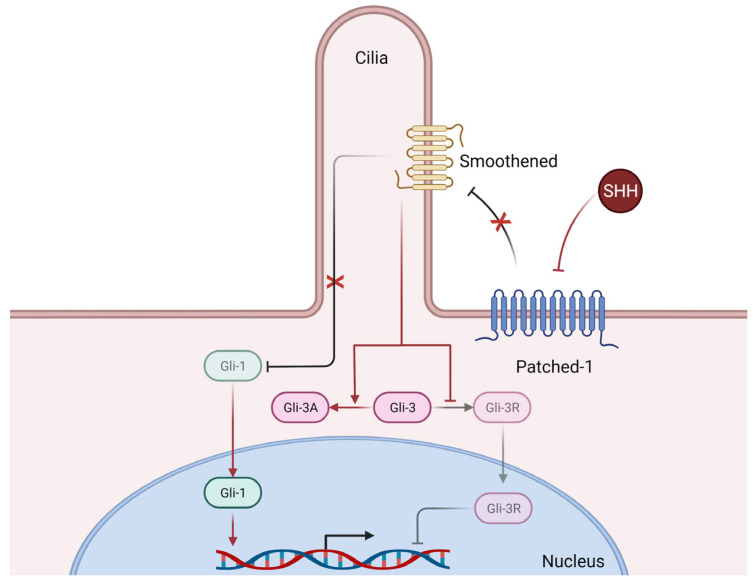
Simplified mechanisms of canonical Hedgehog signaling. Briefly, Sonic Hedgehog (SHH) binds to its receptor Patched-1 thus releasing its inhibition of Smoothened, whose activation in turn releases the previously inhibited Gli-1 transcription factor, who can then translocate to the nucleus and induce the transcription of numerous genes. Furthermore, releasing the inhibition of Smoothened also promotes the expression of the Gli-3 transcription factor in its activator form (Gli-3A) which can then translocate to the nucleus and exert its transcriptional activity (not shown here), whereas in basal conditions (i.e., without SHH), the repressor form (Gli-3R) is the predominantly active form. Of note, Smoothened requires functional primary cilia architecture for functional Hedgehog signaling [31].

The Hedgehog morphogenetic signaling pathway (Figure 3), mainly composed of three ligands, Sonic Hedgehog (SHH), Desert Hedgehog (DHH), and Indian Hedgehog (IHH), three transmembrane receptors (Patched-1, Patched-2, and Smoothened), and three transcription factors (Gli-1, Gli-2, and Gli-3), plays an essential role in the ontogenic development of mammals, but also in adult tissue homeostasis and repair [10].

More specifically, several lines of evidence suggest a key role of the Hedgehog signaling pathway in the physiological and pathological development of the various structures of the eye. For instance, Hedgehog signaling has been extensively studied in the development of the Drosophila’s eye [32] and visual system [33] but also in mammals where it contributes to retinal [34,35,36] and anterior segment development [37,38], including the cornea specifically [34,39]. Notably, abolished Hedgehog signaling in the periocular mesenchyme during ocular development leads to anterior segment dysgenesis conditions, including abnormal corneal dimensions, defective iridocorneal angle, reduced anterior chamber volume, and corneal neovascularization in mice [38]. Furthermore, an in silico whole-exome sequencing genetic analysis of a Han Chinese boy suffering from both anterior segment dysgenesis and morning glory syndrome (a congenital defect of the optic nerve) found compound heterozygous mutations in the *Smoothened* gene (*Smo*), similar to the ocular phenotype of *Smo*-null mice [40].

In adults, the Hedgehog signaling pathway plays a key role in physiological and pathological wound healing. Using a murine splinted excisional wound model, Le et al. showed that the disruption of Hedgehog signaling impacted all aspects of wound healing, namely wound closure, epithelization, granulation formation, vascularity, and proliferation [41]. Further studies have illustrated the importance of Hedgehog signaling in vascularization [42], in pathological wound healing in diabetes [43], in physiological and pathological nerve healing (reviewed in [44]), and more recently in physiological skin regeneration [45].

Finally, Hedgehog pathway has been implicated in the regulation of oxidative stress [11] and autophagy [46], cellular events that are part of the integrated stress response known to participate in numerous ocular diseases including macular degeneration, diabetic retinopathy, cataract, dry eye disease, and keratoconus [47].

All in all, the Hedgehog pathway seems a plausible and pertinent signaling pathway worthy of investigation in the context of corneal wound healing, as reviewed hereafter.

## 4. Distribution of Hedgehog Pathway Effectors in the Ocular Surface

Few studies have investigated the distribution, regulation, and expression of Hedgehog pathway effectors in the ocular surface in adult humans or in animal models.

Expression patterns of Hedgehog pathway effectors have been studied in healthy and wounded corneas in several human diseases (aniridia-related keratopathy [48,49] and diabetic keratopathy [28]) and various animal models of corneal (epithelial debridement [50], trephine-mediated corneal injury [48] or alkali burn injury [51]) or limbal injury (burr-mediated partial limbal injury [52]).

The distribution of Hedgehog pathway readouts in mice, rats, and humans, as reported in the literature, are summarized in Table 1, Table 2 and Table 3, respectively.

### 4.1. Sonic Hedgehog Is Only Expressed in the Wounded Cornea

Expression of Sonic Hedgehog has not been found in healthy corneas but only in injured corneas. Saika et al. found Sonic Hedgehog constitutively expressed in the conjunctival and limbal epithelium but not in the corneal epithelium of healthy rat corneas [50].

Corneal wounding induced transient upregulation of Hedgehog ligands and expression of Gli-3 in the limbus, but also the expression of SHH in the migrating corneal epithelium [50]. Patched-1 upregulation in the edges of the corneal wound was also evidenced [48]. Finally, increased stromal expression of SHH (but not Patched-1) was observed during corneal healing in an alkali-burn rat corneal injury model [51]. This induced expression of Sonic Hedgehog and effectors following corneal injury participates in corneal healing, as detailed in Section 5.

### 4.2. Hedgehog Pathway Effectors Are Expressed in Corneal Endothelial Cells

The expression of hedgehog signaling effectors was also investigated in human donor corneal endothelial cells, where elevated levels of *Shh*, *Gli-1,* and *Gli-2* mRNA were found in the peripheral cornea as compared to the central cornea. Conversely, expression levels of *Smoothened* and *Patched-1* mRNA were similar in peripheral and central cornea. Overall, these results suggest regional variations in Hedgehog signaling activity in the cornea [54].

### 4.3. Hedgehog Pathway Effectors Are Expressed in Limbal Stem Cells

Limbal stem cells constitutively express Hedgehog pathway readouts that participate in limbal stem cell proliferation, as shown in a study of partial limbal injury in mice [52].

Corneal wound induces a transient upregulation of Hedgehog ligands and expression of Gli-3 in the limbus, notably of SHH/Patched-1/Gli-1 signaling within limbal basal cells [50].

As previously mentioned, limbal stem cell proliferation is essential for corneal epithelial renewal as part of the corneal healing process [4].

**Figure 4:**
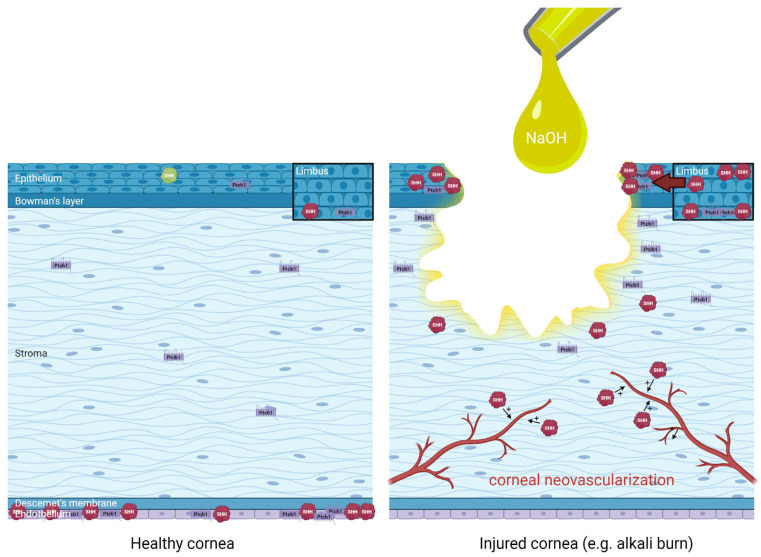
Distribution and regulation of Hedgehog pathway effectors in healthy human cornea (**left** panel) and injured cornea such as following an alkali burn with sodium hydroxide (NaOH) (**right** panel), extrapolating data from animal studies for didactic purposes. An upregulation of limbal Sonic Hedgehog (SHH) in basal epithelial cells and superficial epithelial cells have been evidenced, in association with increased Hedgehog pathway effectors such as Patched-1 (Ptch1) around the wound edges and increased SHH in the migrating epithelium during corneal wound healing. Finally, the role of SHH in the promotion of pathological corneal neovascularization is also illustrated. Distribution based on data from Saika et al. 2004 [50], Fujita et al. 2008 [55], Fujita et al. 2009 [51], Kucerova et al. 2012 [48], Hirata-Tominaga et al. 2013 [54], Fan et al. 2019 [52], Li et al. 2018 [53], Vincente et al. 2018 [49].

The distribution of Hedgehog pathway effectors in healthy and injured cornea (alkali burn) is summarized in Figure 4.

## 5. Current Evidence of Hedgehog Pathway Involvement in Physiological and Pathological Corneal Wound Healing

### 5.1. Implication of Hedgehog Pathway in Physiological Corneal Wound Healing

There is scarce evidence regarding the role of the Hedgehog pathway in physiological corneal wound healing, i.e., in the absence of ocular or systemic conditions affecting the healing process, with only a few studies that have started to address this issue, as reviewed hereafter.

#### 5.1.1. Hedgehog Signaling Promotes Corneal Epithelial Cell Proliferation and Migration during Corneal Wound Healing

In the seminal work by Saika et al., SHH protein was found upregulated in the migrating corneal epithelium following corneal debridement injury in rats, as well as constantly expressed in basal cells of uninjured limbal and conjunctival epithelium, as previously mentioned. Patched-1 was detected in the corneal epithelium and transient nuclear translocation of Gli-3, but Gli-1 was not observed during the healing of the injured corneal epithelium, suggesting autocrine/paracrine regulation of Hedgehog pathway mediated by downstream Gli-3 signaling [50].

Furthermore, in an organ culture of mouse eyes subjected to corneal epithelial debridement, exogenous SHH protein promoted cell proliferation, accompanied by upregulation and nuclear translocation of cyclin D1 (responsible for epithelial cell stemness [56]) in the healing corneal epithelium [50].

Finally, an upregulation of SHH/Gli-3 signaling in the limbal epithelium was also evidenced as early as 2 h post-debridement, when such signaling was still not activated in the migrating corneal epithelium [50]. Such increased signaling was later shown to result in limbal stem cell proliferation [52], essential for corneal epithelial renewal, as previously mentioned.

Topical application of exogenous SHH was shown to significantly improve corneal wound healing rates (22.1 ± 1.2 µm/h vs. 17.9 ±1.4 µm/h in control animals) in a mouse model of trephine corneal injury. This effect required normal PAX-6 concentration (a transcription factor implicated in sensory organ development including the eyes) and a genetic interaction (evidenced using heterozygous PAX-6 and Gli-3-mutant mice) between Hedgehog signaling and PAX-6 in the corneal epithelium [48]. Interestingly, in the same experimental condition, Hedgehog signaling was not required for wound healing. SHH was only expressed in the wounded cornea at very low levels (detectable by RT-PCR but not immunohistochemistry or western blot), similarly to results from Takabatake et al., who detected *Dhh* mRNA but not *Shh* mRNA in mouse corneas [34]. Furthermore, exogenous SHH was shown to directly increase the migratory potential of corneal keratinocytes (using an in vitro Boyden chamber assay). Overall, the authors conclude that Hedgehog signaling pathway is one of several semi-redundant pathways that can stimulate epithelial cell migration but plays only a minor role in the physiological corneal healing response. They posit a model whereby endogenous Hedgehog signaling maintains the corneal epithelium by increasing the mitotic index and the migration potential of epithelial cells, acting via downstream PI3K-Akt- and Gli-mediated signaling pathways [48].

#### 5.1.2. Hedgehog Signaling Promotes Limbal Stem Cell Self-Renewal and Proliferation during Corneal Wound Healing

In a mouse model of partial limbal injury, Fan et al. have shown that intrinsic SHH is crucial for limbal stem cell self-renewal. In such conditions, the activation of Hedgehog signaling promoted limbal stem cell proliferation via Gli-1- and Gli-3-mediated cyclin D1 expression [52]. Interestingly, other signaling molecules implicated in corneal wound healing (see Section 2) such as EGF, PDGF, and IGF have been reported as positive regulators of Gli-1 [52], suggesting a role in modulating Hedgehog signaling in this context.

#### 5.1.3. Hedgehog Signaling Promotes Corneal Epithelial Barrier Homeostasis by Increasing Tight Junction Protein Production

In 2018, Li et al. investigated the role of Ectodyplasin A (EDA) and the mutation of its gene in several tissues, including the cornea. The *Eda* gene regulates the morphogenesis of various ectodermal structures such as hair, teeth, nails, and exocrine glands, and when mutated, is responsible for X-linked hypohidrotic ectodermal dysplasia in humans. In *Eda* mutant *Tabby* mice, tight-junction proteins such as Zonula Occludens-1 (ZO-1) and Claudin-1 were dramatically downregulated, resulting in epithelial barrier dysfunction in various tissues including the cornea [53]. Furthermore, it was shown that EDA promotes corneal epithelial barrier homeostasis via the activation of Hedgehog signaling, which results in an increased production of ZO-1 and claudin-1 [53].

Such Hedgehog-dependent regulation of tight-junction protein synthesis at the cornea level is reminiscent of previous studies that showed the role of Hedgehog pathway inhibition in the disruption of the blood–nerve barrier secondary to endothelial tight-junction protein downregulation in two rat models of spinal [57] and trigeminal [58,59] neuropathic pain.

#### 5.1.4. Hedgehog Signaling Is Necessary for Corneal Endothelial Cell Maintenance

The presence of Hedgehog pathway effectors was investigated in corneal endothelial cells from human donor corneas, as previously mentioned (see Section 4.2). In vitro, SHH was able to induce human donor corneal endothelial cell proliferation. Furthermore, functional Hedgehog signaling was shown to be required for corneal endothelial cell maintenance in this in vitro paradigm [54].

Whether this impacts physiological and/or pathological corneal wound healing remains to be discovered.

### 5.2. Implication of Hedgehog Pathway in Pathological Corneal Wound Healing

As mentioned in Section 4, several studies have investigated the implication of Hedgehog signaling in ocular surface and/or corneal diseases or in pathological corneal healing such as post-injury corneal neovascularization, shedding additional light on the possible roles of such signaling pathway in corneal health and disease.

#### 5.2.1. Hedgehog Signaling in Aniridia-Related Keratopathy

The activation of Hedgehog signaling pathway (evidenced by increased Gli-1 production) was observed in aniridia-related keratopathy (ARK) patients but not healthy corneas, where it was suggested to play a role in the cell proliferation changes observed in the epithelium and subepithelial pannus of ARK corneas via increased HES-1 signaling [49].

Furthermore, as previously mentioned, in a mouse model of trephine-mediated corneal injury, exogenous topical application of SHH facilitated corneal wound healing, but only in wild type animals (healthy corneas) and not in PAX-6^+/−^ animals (a murine model of aniridia), suggesting that pharmacological modulation of Hedgehog signaling may promote wound healing in some corneal diseases, such as those resulting from injury or infection, but not in human aniridia (a genetic condition secondary to PAX-6 mutation) [48].

#### 5.2.2. Hedgehog Signaling in Diabetic Keratopathy

Diabetic keratopathy is a sight-threatening corneal disease composed of several symptomatic conditions including delayed epithelial wound healing, recurrent erosions, and sensory nerve neuropathy, the latter being implicated in delayed epithelial wound healing, as shown in a recent study by Zhang et al. [28], detailed hereafter.

In naïve mice, corneal denervation by resiniferatoxin severely impaired corneal wound healing and markedly upregulated proinflammatory gene expression (Interleukin-1β (IL-1β), C-X-C Motif Chemokine ligand 2 (CXCL2), and Nitric Oxide Synthase 2 (NOS-2)), whereas topical administration of neuropeptides CGRP, SP, and VIP partially reversed the effects of resiniferatoxin. Furthermore, VIP specifically increased the corneal expression of anti-inflammatory cytokine IL-10 [28].

Moreover, following trephine-mediated corneal wounding, VIP and VIP type 1 receptor (VIPR1) expression increased in normal corneas but not in corneas of diabetic mice. Pharmacological inhibition of VIPR1 in normal corneas attenuated corneal wound healing, dampened wound-induced expression of neurotrophic factors, and exacerbated inflammatory responses, whereas the administration of exogenous VIP had opposing effects in corneas of diabetic mice [28].

Interestingly, pharmacologically downregulating SHH expression in normal corneas decreased the rate of corneal wound healing, whereas exogenous topical administration of SHH in corneas of diabetic mice increased the rate of corneal wound healing. Furthermore, inhibition of SHH signaling dampened VIP-promoted corneal wound healing, suggesting that VIP regulates corneal epithelial wound healing, inflammatory response, and nerve regeneration in an SHH-dependent manner [28].

#### 5.2.3. Role of Hedgehog Signaling in Post-Injury Corneal Neovascularization

Although neovascularization is beneficial for tissue repair, the cornea must remain avascular to maintain transparency and proper light refraction. Injury-induced neovascularization in the cornea is thus an unfavorable outcome leading to impaired vision [51].

The potential role of Hedgehog signaling in neovascularization was investigated using a co-culture system of human vein endothelial cells (HUVECs) and human fibroblasts. Recombinant SHH accelerated vessel-like tube formation by HUVECs, suggesting a role of SHH in neovascularization, which was subsequently shown to be VEGF-independent [51].

Furthermore, SHH upregulation was detected until day 20 in the healing stroma of an alkali-burn neovascularized mouse cornea, suggesting that SHH could participate in the development of neovascularization in the healing cornea [51]. Implantation of a SHH-containing polymer pellet in the corneal stroma induced marked neovascularization in the stroma from limbal vessels, whereas conversely, a cyclopamine-containing pellet (a Hedgehog pathway inhibitor) reduced neovascularization (as compared to controls), confirming the role of endogenous SHH in the promotion of corneal neovascularization [51].

Consequently, a pharmacological blockade of Hedgehog signaling might have a beneficial inhibitory effect of neovascularization development in the cornea during healing. Indeed, as shown by the same authors, topical cyclopamine suppressed the development of peripheral neovascularization following cauterization of the central cornea of rats [51].

## 6. Future Research and Perspectives

Apart from the implication of Hedgehog signaling in physiological and pathological corneal wound healing shown in the aforementioned studies (that will require further confirmation and exploration of other players such as immune cells), other research avenues could be explored, investigating the possible role of Hedgehog signaling in various corneal diseases.

Indeed, considering the existing data implicating the Hedgehog pathway in the pathophysiology of other similar diseases/pathological processes, its involvement could be hypothesized in several corneal diseases, as discussed hereafter.

### 6.1. Could Hedgehog Signaling Be Implicated in Benzalkonium Chloride-Mediated Ocular Toxicity?

Benzalkonium chloride (BAK) is a commonly used preservative in about 70% of eye drop solutions [60], whose deleterious effects related to its local toxicity have been brought to attention these past few years [61,62,63]. From a clinical standpoint, such toxicity can be a source of iatrogenic aggravation of preexisting ocular diseases (for which the eyedrops where initially prescribed) such as in dry eye disease [63].

Although the ocular toxicity of BAK has been well-studied [64,65,66], the underlying pathophysiological mechanisms are still not fully understood [60].

In a rabbit cornea study, the intraocular pressure-lowering agent latanoprost with 0.02% benzalkonium chloride (BAK) significantly increased corneal epithelial permeability (assessed by carboxyfluorescein uptake) compared to travoprost (without BAK preservative) or vehicle. This increased permeability was associated with significant loss of (unspecified) tight-junction proteins in the latanaprost group, most probably related to the presence of BAK [67].

More recently, Zhang et al. showed that the topical administration of 0.05% and 0.1% BAK dose-dependently disrupted corneal endothelial cell morphology, altered Connexin 43 (Cx43) expression and Zonula Occludens 1 (ZO-1) distribution, and reduced Cx43 expression in the cornea of albino rabbits. This was associated with reduced gap-junction intercellular communication activity [68].

Interestingly, as mentioned previously, Hedgehog signaling has been implicated in corneal epithelial barrier maintenance (see Section 5.1.3), whereas the inhibition of Hedgehog signaling has been shown to mediate increased blood–nerve barrier permeability in injured nerves by downregulating tight-junction protein expression at the endothelial cell level [57,58,59]. Furthermore, as BAK is a quaternary ammonium compound that has been shown to destabilize and solubilize cellular lipid membranes [69], and considering the necessity of a normal ciliary architecture within the cell’s lipid membrane for functional Hedgehog signaling [31], one can easily hypothesize that, by destabilizing the lipid membrane, BAK could lead to loss of functional Hedgehog signaling that could account for some (or even most) of its toxic effects.

Finally, BAK has been shown to be neurotoxic and reduce corneal nerve fiber density and neurite outgrowth [70], both mechanisms related to dysfunctional Hedgehog signaling (as reviewed in [44]).

### 6.2. Could Hedgehog Signaling Be Implicated in the Pathophysiology of Neurotrophic Keratopathy?

Neurotrophic keratopathy is a condition associating corneal epitheliopathy with frank epithelial defects and reduced or absent corneal sensations (reviewed in [71]). It is believed that the corneal alterations are secondary to the loss of trophic support from corneal nerves that degenerate secondary to proximal trigeminal nerve injury [71,72]. Such trophic support in physiological conditions is thought to result from epithelial-nerve crosstalks (as mentioned in Section 2) under the control of neurotrophic factors such as GDNF, BNDF, or NGF, to name a few.

Considering the role of Hedgehog signaling in the production of such neurotrophic factors in the context of peripheral nerve healing (reviewed in [44]), this pathway could also participate in the pathophysiology of neurotrophic keratopathy. Investigating this possibility could allow the development of new therapeutic strategies to simultaneously promote both corneal epithelium and corneal nerve healing, notably by topical applications of Hedgehog pathway modulators, as proposed in other clinical situations [55].

## 7. Conclusions

The morphogenetic Hedgehog signaling pathway participates in corneal wound healing mainly at the level of the corneal and limbal epithelium, where Sonic Hedgehog-mediated signaling promotes limbal stem cell proliferation and corneal epithelial cell proliferation and migration following corneal injury. Furthermore, as a morphogenetic pathway constitutively expressed in adult tissues, Hedgehog signaling could also participate in corneal epithelial barrier homeostasis. Conversely, Hedgehog signaling could participate in deleterious aspects of corneal healing such as corneal injury-related neovascularization.

By gaining a better understanding of the role of this double-edged sword in physiological and pathological corneal wound healing, fascinating new research avenues and novel therapeutic strategies will undoubtedly emerge.

## Figures and Tables

**Table 1:** Distribution of Hedgehog pathway effectors in the ocular surface of mice (DHH = Desert Hedgehog, GSK-3 = Glycogene Synthase Kinase 3, HPRT = Hypoxantine-guanine PhosphoRibosylTransferase, PAX-6 = Paired Box Protein 6, SHH = Sonic Hedgehog).

Structure	Tissue	Hedgehog Pathway Effector(s)	Expression Pattern	Reference(s)
**Cornea**	Whole cornea	DHH, Gli-2, Gli-3, Patched-1, Sufu, PAX-6, GSK-3, Smoothened, HPRT	Constitutive	[48]
		SHH	Induced following injury	[48]
	Epithelium	*Shh* mRNA	Induced (227-fold upregulation in a healing alkali-burned neovascularized cornea	[51]
		SHH	Induced in a healing epithelium until day 20	[51]
		Patched-1	Expressed at the wound edge	[48]
	Stroma	SHH	Induced in a healing stroma until day 20	[51]
**Limbus**	Limbal stem cells	Patched-1 Smoothened Gli-1 Gli-3 Gli-3R ^1^	Constitutive	[52]

^1^ Repressor form of Gli-3.

**Table 2:** Distribution of Hedgehog pathway effectors in the ocular surface of rats (SHH = Sonic Hedgehog).

Structure	Tissue	Hedgehog Pathway Effector(s)	Expression Pattern	Reference(s)
**Cornea**	Epithelium	Patched-1	Low constitutive	[50]
		SHH	Transient induction with a peak at 12 h post-debridement in the migrating corneal epithelium	[50]
**Limbus**	Basal epithelial cells	SHH, Patched-1	Constitutive	[50]
	Epithelium	SHH	Transiently induced at 2 h following debridement	[50]
**Conjunctiva**	Basal epithelial cells	SHH, Patched-1	Constitutive	[50]

**Table 3:** Distribution of Hedgehog pathway effectors in the ocular surface of humans (EC = Endothelial cells, HES-1 = Hairy and Enhancer of Split 1, SHH = Sonic Hedgehog).

Structure	Tissue	Hedgehog Pathway Effector(s)	Expression Pattern	Reference(s)
**Cornea**	Epithelium	*Shh* mRNA *Gli-1* mRNA	High induced ^1^	[53]
		Gli-1 HES-1	High induced ^2^	[49]
	Endothelial cells	*Shh, Smoothened, Patched-1*, *Gli-1*, *Gli-2* mRNA	Constitutive Higher expression in peripheral vs. central EC for *SHH, Gli-1* and *Gli-2* mRNA	[54]
**Limbus**	Basal epithelial cells	SHH	Low constitutive	[55]
**Bulbar** **conjunctiva**	Basal epithelial cells	SHH	Low constitutive	[55]
**Palpebral** **conjunctiva**	Basal epithelial cells	SHH	Low constitutive	[55]

^1^ Induction by ectodysplasin A in human corneal epithelial cells. ^2^ Evidenced in aniridia-related keratopathy.

## Data Availability

Not applicable.

## Abbreviations

ARK	Aniridia-Related Keratopathy
BAK	Benzalkonium Chloride
BDNF	Brain-Derived Neurotrophic Factor
CCL-2	C-C Motif Chemokine Ligand 2
CGRP	Calcitonin Gene-Related Peptide
Cx43	Connexin 43
CXCL2	C-X-C Motif Chemokine Ligand 2
DHH	Desert Hedgehog
EC	Endothelial Cells
EDA	Ectodysplasin A
EGF	Epidermal Growth Factor
EMMPRIN	Extracellular Matrix MetalloPRoteinase Inducer
GDNF	Glial cell Derived Neurotrophic Factor
GSK-3	Glycogen Synthase Kinase 3
HES-1	Hairy and Enhancer of Split 1
HGF	Hepatocyte Growth Factor
HPRT	Hypoxanthine-guanine PhosphoRibosylTransferase
HUVEC	Human Umbilical Vein Endothelial Cells
IGF	Insulin-like Growth Factor
IL-1	Interleukin 1
IL-1β	Interleukin 1β
IL-10	Interleukin 10
IHH	Indian Hedgehog
KGF	Keratinocyte Growth Factor
MMP-1	Matrix MetalloProteinase 1
MMP-2	Matrix MetalloProteinase 2
MMP-3	Matrix MetalloProteinase 3
MT1-MMP	Membrane-type 1-Matrix MetalloProteinase
NGF	Nerve Growth Factor
NOS-2	Nitrix Oxide Synthase 2
PACAP	Pituitary Adenylate Cyclase-Activating Polypeptide
PAX-6	PAired boX protein 6
PDGF	Platelet-Derived Growth Factor
PI3K	Phosphoinositide 3-Kinase
SHH	Sonic Hedgehog
SP	Substance P
TGF-β	Transforming Growth Factor β
TNF-α	Tumor Necrosis Factor α
VEGF	Vascular Endothelial Growth Factor
VIP	Vasoactive Intestinal Peptide
VIPR1	Vasoactive Intestinal Peptide Receptor 1
ZO-1	Zonula Occludens 1
